# Automated detection of activity onset after postictal generalized EEG suppression

**DOI:** 10.1186/s12911-020-01307-7

**Published:** 2020-12-24

**Authors:** Bishal Lamichhane, Yejin Kim, Santiago Segarra, Guoqiang Zhang, Samden Lhatoo, Jaison Hampson, Xiaoqian Jiang

**Affiliations:** 1 Electrical and Computer Engineering Department, Rice University, 6100 Main St, Houston, TX USA; 2 School of Biomedical Informatics, UT Health, 7000 Fannin St Suite 600, Houston, TX USA; 3 Department of Neurology, McGovern Medical School, UT Health, 6430 Fannin St, Houston, TX USA

**Keywords:** PGES, SUDEP, Epilepsy, EEG suppression

## Abstract

**Background:**

Sudden unexpected death in epilepsy (SUDEP) is a leading cause of premature death in patients with epilepsy. If timely assessment of SUDEP risk can be made, early interventions for optimized treatments might be provided. One of the biomarkers being investigated for SUDEP risk assessment is postictal generalized EEG suppression [postictal generalized EEG suppression (PGES)]. For example, prolonged PGES has been found to be associated with a higher risk for SUDEP. Accurate characterization of PGES requires correct identification of the end of PGES, which is often complicated due to signal noise and artifacts, and has been reported to be a difficult task even for trained clinical professionals. In this work we present a method for automatic detection of the end of PGES using multi-channel EEG recordings, thus enabling the downstream task of SUDEP risk assessment by PGES characterization.

**Methods:**

We address the detection of the end of PGES as a classification problem. Given a short EEG snippet, a trained model classifies whether it consists of the end of PGES or not. Scalp EEG recordings from a total of 134 patients with epilepsy are used for training a random forest based classification model. Various time-series based features are used to characterize the EEG signal for the classification task. The features that we have used are computationally inexpensive, making it suitable for real-time implementations and low-power solutions. The reference labels for classification are based on annotations by trained clinicians identifying the end of PGES in an EEG recording.

**Results:**

We evaluated our classification model on an independent test dataset from 34 epileptic patients and obtained an AUreceiver operating characteristic (ROC) (area under the curve) of 0.84. We found that inclusion of multiple EEG channels is important for better classification results, possibly owing to the generalized nature of PGES. Of among the channels included in our analysis, the central EEG channels were found to provide the best discriminative representation for the detection of the end of PGES.

**Conclusion:**

Accurate detection of the end of PGES is important for PGES characterization and SUDEP risk assessment. In this work, we showed that it is feasible to automatically detect the end of PGES—otherwise difficult to detect due to EEG noise and artifacts—using time-series features derived from multi-channel EEG recordings. In future work, we will explore deep learning based models for improved detection and investigate the downstream task of PGES characterization for SUDEP risk assessment.

## Background

Epilepsy is a neurological disorder where a person has recurrent abnormal electrical activities in the brain (*seizures*). Even though epilepsy is widely prevalent [1], a good understanding of its causes remains elusive in up to 50% of the cases [2]. The cases of SUDEP (sudden unexpected death in epilepsy), a condition where an epileptic patient has sudden death without any specific known cause leading to an unignorable rate of epilepsy death (8-17%), remain even more unfathomable. This markedly contrasts with the associated clinical importance, since SUDEP is the cause of premature death in about 1 in 1000 patients with epilepsy [3]. A line of thought for SUDEP is that the brain activity aberrations result in cardiovascular and respiratory dysfunctions, which then lead to death [4]. However, the cases of SUDEP are highly heterogeneous, appearing in contrasting cases and conditions. For instance, while most of the SUDEP cases are found to be related to a preceding seizure event, there are still some other reported cases of SUDEP without an immediately preceding seizure [5]. Complete pathophysiology of SUDEP still remains uncertain to this date.

Though SUDEP cases are heterogeneous, some biomarkers have been found to be associated with the SUDEP risk [6–10]. One such biomarker being actively investigated is *postictal generalized EEG suppression* (PGES). As the name suggests, PGES is a suppression of brain activity occurring immediately (within 30 s) after a seizure. The suppression is commonly defined to have occurred when all the EEG channels have activity of less than 10 $\upmu V$, after properly accounting for noise and artifacts [11, 12]. After the suppression, there is a return of normal EEG activity or likely a state of slow wave activity [12]. One of the earlier works investigating the relation between PGES and SUDEP risk were done by the authors in [12]. The authors found increased occurrence of PGES in patients with SUDEP, compared to a control group. Similarly, increased duration of PGES was found to be directly related to SUDEP risk. Nonetheless, there have been some other studies that could not establish a similar relation between increased PGES duration and higher SUDEP risk. The study in [11] found that neither the presence of PGES nor its duration were related to SUDEP risk. In this context, the authors in [13] observed that this reported inconsistency could be due to the specific nature of the cohort in the latter study. All the included patients presented temporal lobe epilepsy and were undergoing standard pre-surgical evaluations. In another study [14], the authors found PGES to be of rather shorter duration in patients with SUDEP, compared to the control group. However, it was also noted by the authors that they had difficulty in identifying the end of PGES. Multiple occurrences of closely located suppression episodes were seen. When the end of the last suppression episode was considered for the total duration of PGES, the patients with SUDEP then had longer PGES (though the difference was not significant). As evident from this study, detecting the end of PGES is complex and this complexity has impact on assessing the relation between PGES and SUDEP risk. Overall, though the exact nature of this relation remains contested, the general relevance of PGES for SUDEP and the need for further investigation has been well recognized [15–19]. Given the reported difficulty in detecting PGES, it would be desirable to provide an *automated yet robust method for PGES detection* to assist investigations relating PGES to SUDEP risk.

**Fig. 1:**
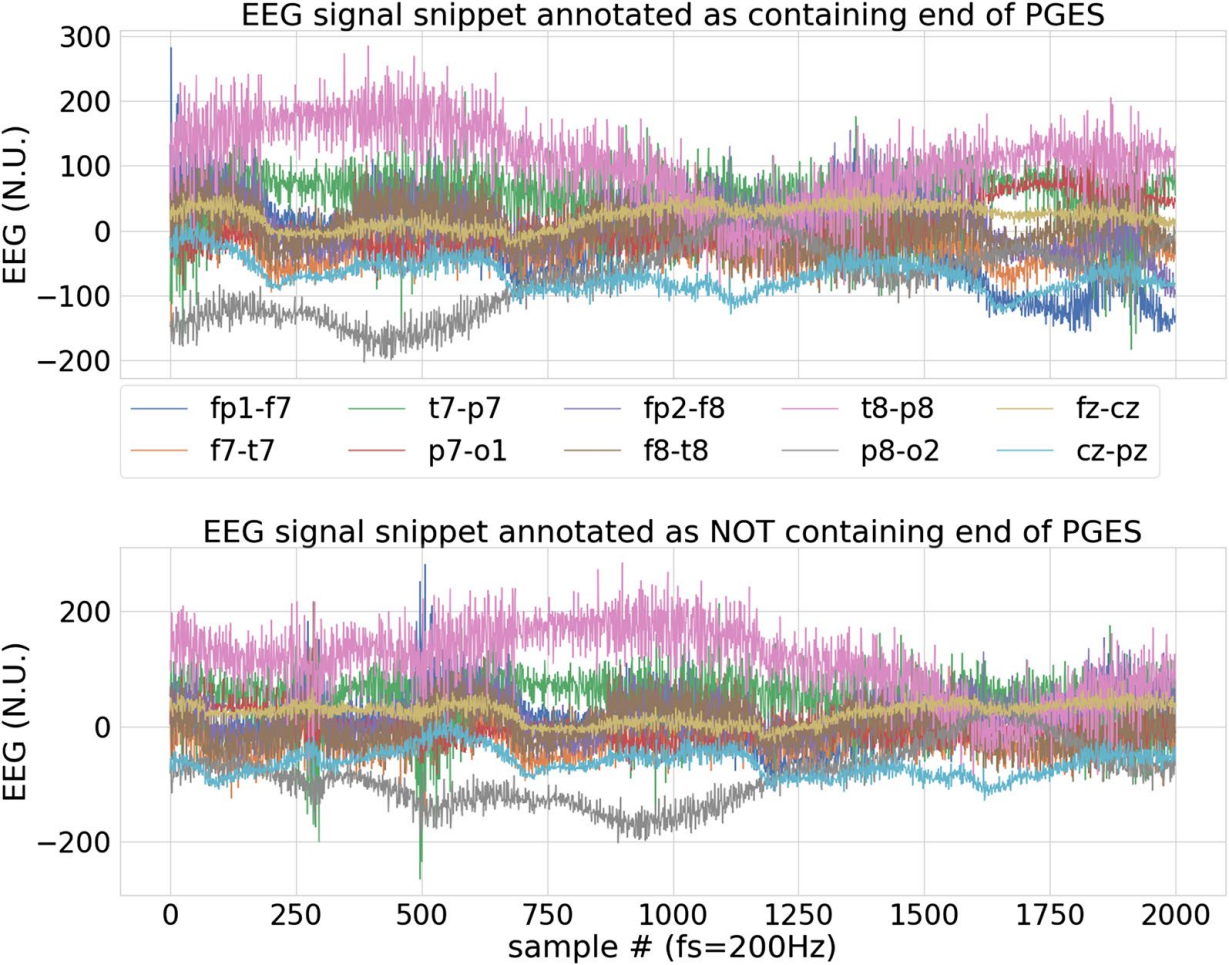
SUDEP EEG pattern. An example of an EEG signal snippet annotated by a clinician as containing the end of PGES (top), and not containing any such PGES state transition (bottom). The differentiation can only be made by experienced clinical professionals after thorough and contextual assessment of the recordings. These assessments are still often done only retrospectively (with the assistance of video). A real-time automated detection of PGES state transition will enable PGES characterization for SUDEP risk assessment

A standard definition of PGES has been commonly used to identify its onset. As stated earlier, a general suppression in EEG activity (less than 10 $\upmu V$ in all the EEG channels) occurring immediately after the seizure marks the onset of PGES. However, detecting the end of PGES can be problematic due to noise and artifacts from various sources (i.e., movement, sensor error, breathing, etc.) This was for example acknowledged in the work of [20] for automated detection of PGES. In their work, features derived from the energy in different frequency bands of the EEG signal were used to classify if a given EEG segment was PGES. The authors noted that the identification of the end of PGES is very challenging, even for trained clinicians. Proper judgment has to be made, otherwise, a mere artifact could be detected as transition out of PGES. An example of an EEG signal snippet annotated by a clinician identifying the end of PGES is shown in Fig. 1. As it can be seen, detecting the end of PGES exclusively from visual features is not trivial as EEG recordings are inevitably noise-prone. A trained clinician is usually relying on his/her experiences, information in multiple channels, and other contextual information (e.g. patient’s history) when doing a retrospective assessment for PGES. As transition out of PGES could be confused with aberrations due to noise and artifacts, the authors in [20] used heuristics to get rid of false detection of the end of PGES. The difficulty in correctly identifying the end of PGES could also have been a reason for the authors in [14] to have observed multiple PGES occurring close to each other (which might rather just have been a single period of PGES). A robust system for identifying the end of PGES, in conjuction with PGES onset detection, will lead to accurate characterization of the PGES and SUDEP risk assessment.

**Fig. 2:**
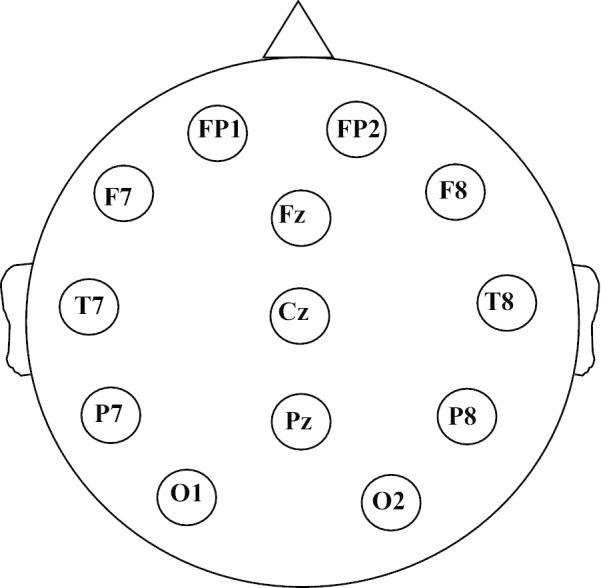
Recording setup. 13 EEG electrodes used for the recordings in our dataset. The obtained signals are processed to obtain 10-channel bipolar EEG montages. These channels are: FP1–F7, F7–T7, T7–P7, P7–O1, FP2–F8, F8–T8, T8–P8, P8–O2, Fz–Cz, and Cz–Pz

**Fig. 3:**
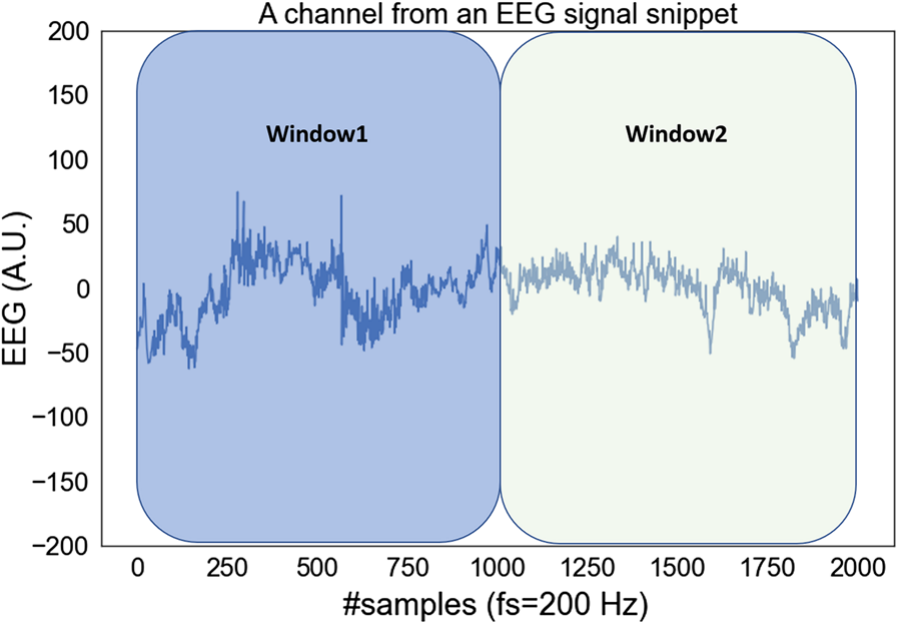
Temporal signal ratio features. An example showing how the temporal signal ratio features are computed for an EEG channel. The ratio of statistics (such as mean) of the signal in the last half (Window2 here) to that in the first half (Window1 here) is taken as a feature. The temporal signal ratio features are computed for each EEG channel. Additional features summarizing the ratio features across the channels are also computed

**Fig. 4:**
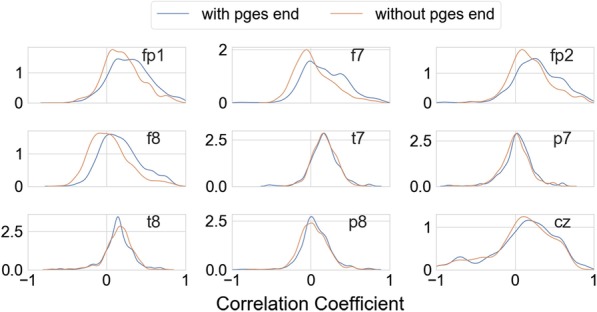
Feature distribution. Distribution of correlation based features in the two classes: snippets with and without the end of PGES. The features shown represent the correlation of the signal in the Fz channel with signals in all other channels

In this work, we investigate the feasibility of detecting the end of PGES using EEG signal features. The features were constrained to be computationally inexpensive, thus suitable for real-time and low-power solutions. We trained a classification model that can identify if a given EEG signal snippet consists of an end of PGES. The model was trained using recordings obtained from 134 patients with epilepsy and evaluated in an independent test set consisting of recordings from 34 epileptic patients. Recently, the authors in [21] have also proposed a system for automatic PGES end detection using a diverse set of EEG signal features. In contrast to the work in [21], we use only temporal signal features and correlation features which are computationally cheap. Further, we consider the possibility of PGES transitioning immediately to normal EEG pattern (instead of slow wave activity only) [20] and thus retain a wider frequency band of EEG signals for analysis. Additionally, we employ patient-independent evaluation for PGES end detection which reflects the clinical deployment scenario of model evaluation on unseen patient’s data. Finally, in this work, we also evaluate the contribution of different EEG channels for the task of PGES end detection which gives some insights on optimal measurement setup for SUDEP monitoring systems.

## Methods

### SUDEP dataset

The analysis in this work was done using the SUDEP dataset. The patient cohort information is described in the hackathon editorial paper. The SUDEP dataset consists of EEG recordings with 13 electrodes (Fig. 2), with signals obtained at varying sampling frequencies of 200–250 Hz. All the recordings were converted to a sampling frequency of 200 Hz using frequency re-sampling. EEG recordings are processed to obtain 10-channel bipolar montages. These bipolar montages consists of: Fp1–F7, F7–T7, T7–P7, P7–O1, Fp2–F8, F8–T8, T8–P8, P8–O2, Fz–Cz, and Cz–Pz. In this work, we refer to a channel in the bipolar montage by the name of its first electrode for brevity (e.g. FP1–F7 will be referred as FP1). The recordings in the SUDEP dataset were annotated by medical experts, marking the timing of the end of PGES. The SUDEP dataset is separated into a training records set (for developing models and algorithms) and a test dataset (for independent validation). The training records set consist of recordings from 134 patients with epilepsy. Average duration of the EEG recordings in the training records set, which were obtained from peri-ictal period for each patient, is $46.90 \pm 33.04$ s. The test dataset is derived from a separate group of 34 epileptic patients. The dataset consists of 12,345 short EEG snippets, each 10 s in length corresponding to the maximum latency for detecting the end of PGES. Of the 12,345 snippets, 3219 snippets contain the end of PGES. We posed the problem of detecting the end of PGES as a classification task i.e. to classify whether a given snippet includes an end of PGES or not. We trained a classification model using a training dataset obtained from the training records in the SUDEP dataset as described next.

**Fig. 5:**
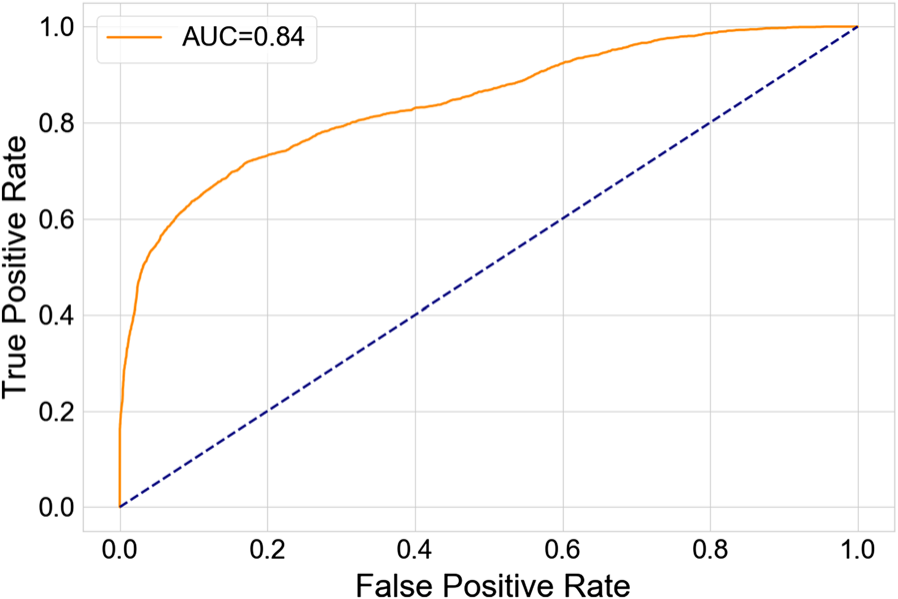
ROC for PGES end detection. ROC curve for detecting the end of PGES in the test set of the SUDEP dataset for a random forest classifier

**Fig. 6:**
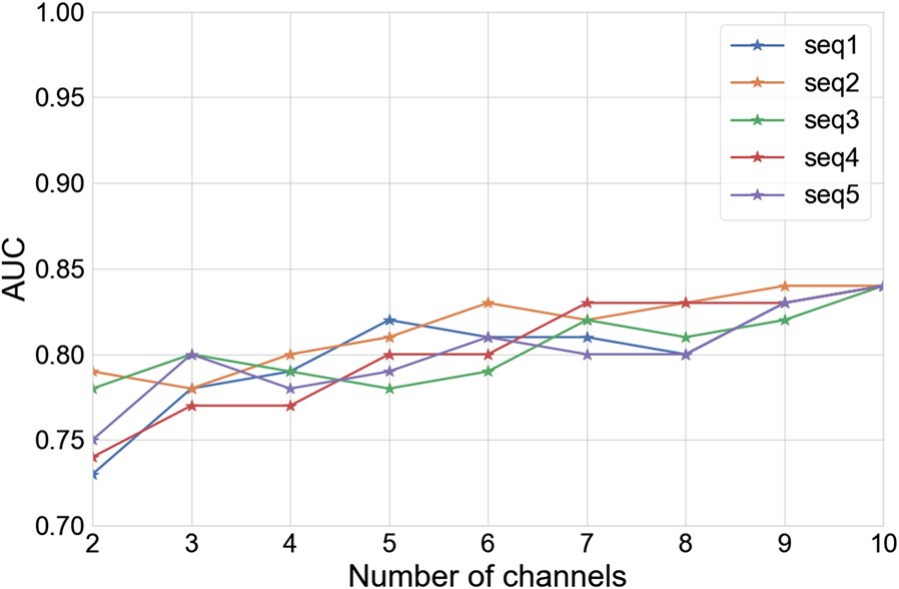
Performance with different channel combinations. AUC obtained with increasing number of channels, for five different instances of EEG channel sequences. Channel combinations from two (minimum required for feature definition) to ten (all channels) are considered

### Training dataset

We used the raw EEG records (provided for training) to create a customized training dataset consisting of several EEG snippets, each 10 s in duration (corresponding to the desired maximum latency for detecting the end of PGES). These EEG snippets were extracted by placing a 10-s signal extraction window at a random time-point of a given recording. An extracted EEG snippet is of positive class (i.e. containing the end of PGES) if it includes the annotated location of transition from PGES to EEG activity. We guided the snippet extraction process to have similar number of snippets from each patient in the training dataset, and also roughly equal representation of both classes (snippets with and without the end of PGES). For our analysis, we prepared a training dataset consisting of 6241 snippets (of which 3318 contained the end of PGES).

### Feature extraction

Signal features were extracted from each EEG snippet and provided as input to a classification method. However, EEG recordings can be corrupted by noise and artifacts, see e.g., the noisy signals in Fig. 1. Hence, we first denoised the signal in each channel of the EEG snippet using a 5th order Butterworth bandpass filter. Filter implementation from Scipy library [22] was used and a forward-backward filtering was applied to avoid phase delay. The passband for the filter was set to 1–47 Hz, retaining most of the information of an EEG signal [23] while suppressing noise in frequencies outside this band. We extracted highly predictive but computationally inexpensive features from the time series of the EEG signals based on correlations and temporal ratio.

#### Correlation features

The end of PGES (and subsequent transition to EEG activity) is likely a significant event in comparison to other brain activity events, appearing strongly in most of the EEG channels. Thus, correlation features could be relevant for our classification task. We computed inter-channel correlation coefficients (Pearson correlation coefficients) from each EEG snippet as the correlation features. This feature set is a simple way to characterize the relation between signals in different EEG channels. The dimension of the correlation features is 45 (all possible channel pairs chosen from the 10 channels).

#### Temporal signal ratio

The transition from PGES to onset of EEG activity is a temporal change of state. Thus, we extracted temporal signal ratio based features to characterize temporal changes in a signal. Temporal signal ratio is obtained from the ratio of signal statistics in the last half of a given time-series to the signal statistics in the first half. An example showing how this feature is computed is represented in Fig. 3. We used mean and variance as the signal statistics and computed a total of 24 features from temporal signal ratio. These features are listed below.

- *Temporal mean ratio* 10 features for each EEG channel capturing the ratio between the mean of the signal in the last half and the mean in the first half.
- *Temporal variability ratio* 10 features capturing the ratio between the variance of the signal in the last half and the variance in the first half.
- *Temporal mean ratio summary* 2 features were computed as summary of the temporal mean ratio features. For each channel, its temporal mean ratio feature was first normalized by the signal variance computed from the entire time-series. Then the sum of the normalized feature across the channels was computed as one of the summary features. The second summary feature was the normalized temporal mean ratio corresponding to the channel with least signal variance. The criterion of least variance has been used as an indication for the channel less contaminated by noise and artifacts.
- *Temporal variability ratio summary* Counterpart of the temporal mean ratio summary features but computed based on the temporal variability ratio features.

#### Low-frequency EEG temporal signal ratio

We computed 24 more features analogous to the temporal signal ratio features described, with the only difference being that the EEG signal was first filtered to retain only the low frequency components. We used a 5th order Butterworth bandpass filter with cutoff of 3 Hz and 8 Hz. Explicit characterization of the lower frequency in the EEG signal was done with this feature set to get a better representation of the slow wave activities. Previous studies have shown that the transition out of PGES is often detectable in the slow wave activities [12]. Very low frequencies (<3 Hz) were avoided as these frequency bands are likely to be strongly affected by noise and artifacts such as drifts.

#### Sliding signal difference

To characterize the temporal signal dynamics with a higher resolution, we computed sliding signal difference based features from the low-frequency EEG signal (3–8 Hz). These features were computed as follows: A window corresponding to 50 samples (0.25 s), with a step size of 10 samples, was run over each EEG channel. Within a window, the difference of the sum of signals in the last half of the window (i.e. 25 samples) with the sum of signals in the first half of the window was calculated. Then, from all the difference values computed for each step, the maximum was retained as the feature for a given channel. 10 features were obtained corresponding to the 10 EEG channels. Sum of the features across the channel, normalized by the corresponding signal variance, was also computed as a summary feature. Finally, the normalized feature for the channel with the least signal variance was also retained as a separate feature. Thus, a total of 12 features were computed.

#### Low-frequency EEG signal features

While signal ratio features characterize the signal dynamics over time, we also extracted other global features from the low-frequency EEG signal. Particularly, we computed the mean and variance of the low-frequency EEG signal for each channel. This results in 20 features. We also computed the sum of the means across the channels, after normalizing each mean by the variance of the corresponding signal. Moreover, the normalized mean of the channel with the least variance was also retained as a separate feature. Thus, a total of 22 feature were computed as low frequency EEG signal features.

Overall, each input EEG snippet was characterized by 127 features as summarized in Table 1. These features were then used in a classification framework for identifying if a given snippet contains the end of PGES or not.

**Table 1:** Features computed from EEG signal time-series

Feature set	Number of features
Correlation features	45
Temporal signal ratio	24
Low-freq EEG temporal signal ratio	24
Low-freq EEG signal features	22
Sliding signal difference	12
Total	127

A total of 127 features are extracted based on inter-channel correlation and intra-channel temporal signal dynamics

### Classification and evaluation metric

We implemented a random forest (random forest (RF)) model for classification. RF is an ensemble of several decision tree classifiers built using a random subset of features on a sample of the training dataset. The number of trees was set to 501 based on cross-validation in the training dataset. We selected RF as the machine learning model because: it is less prone to overfitting, compared to the constituent tree classifiers, due to ensemble averaging [24]; it has only a few sensitive hyperparameters to tune and is thus easier to train [25]; it works well even with features of different scales; and it has been found to perform better than many other classifiers in large empirical benchmarks [26, 27]. For evaluating the trained classifier, we adopted the area under the curve (AUC) of the receiver operating characteristic (ROC) curve obtained from the test dataset.

## Results

Each EEG snippet was characterized by 127 features derived from the time-series of the signal. In Fig. 4, we show an example of correlation based features computed for the snippets in the training dataset. Feature distribution in the two classes (snippets with and without the end of PGES) is presented. For brevity, only the features obtained from the correlation of the Fz EEG channel with other channels are shown in the example. The mean correlation for data in the negative class (snippets without the end of PGES) was 0.10 (± 0.24) while that for data in the positive class (snippets with the end of PGES) was 0.16 (± 0.26). We trained a random forest classifier with the EEG snippets in the training dataset. The trained classifier was then evaluated using the test dataset. The ROC curve obtained for this evaluation is shown in Fig. 5, yielding an AUC of 0.84. The corresponding precision, recall, and F1-score were 0.54, 0.74, and 0.63 respectively.

While the ROC curve presented in Fig. 5 shows the performance of the classifier trained using the entire feature set, we also evaluated classifiers trained with only subsets of the full feature set. The subsets that have been evaluated are based on feature type (e.g. all correlation based features, all features from temporal signal ratio, and so on). The results obtained for evaluation with different feature subsets are shown in Table 2.

**Table 2:** ROC AUC obtained with different feature subsets

Feature Set	AUC
Correlation features (45 features)	0.77
Temporal signal ratio (24 features)	0.72
Low-freq EEG temporal signal ratio (24 features)	0.72
Low-freq EEG signal features (22 features)	0.79
Sliding signal ratio (12 features)	0.73

To compare the effect of EEG signal noise on the downstream classification task, we evaluated the classification pipeline with and without the noise filter (bandpass filter between 1 and 47 Hz). The results obtained are shown in Table 3. This evaluation was done with a feature set consisting of correlation features and temporal signal ratio features only, since low-frequency features implicitly filter the noise in frequencies outside of the passband (3–8 Hz).

**Table 3:** Effect of EEG noise on the classification task

Feature set	AUC
With noise filter	0.80
Without noise filter	0.73

PGES end detection is evaluated using correlation and temporal signal features, computed with and without a noise filter

Further, we evaluated the classification results using channels in certain brain regions (using EEG nomenclature) only. The results obtained for classification using features derived from the EEG channels in the left (FP1, F7, T7, P7), right (FP2, F8, T8, P8), and the central (Fz, Cz) regions are given in Table 4. We also evaluated the classification results when using a channel combination representing a diametric region (FP1,P8).

**Table 4:** PGES end detection using different subsets of the EEG channels

Region	AUC
Left (4 channels)	0.78
Right (4 channels)	0.75
Center (2 channels)	0.79
Diametric (2 channels)	0.68

Given that we have multi-channel EEG recordings, we also evaluated the impact of having limited number of channels. We compute classification results obtained with increasing number of channels, from two channels (minimum required for feature definition) to ten channels (all the channels). This experiment was conducted with different sequence of EEG channels. E.g. one sequence could be {(FP1), (FP1, F7), $\ldots$, (FP1, F7, T7, P7, FP2, F8, T8, P8, Fz, Cz)} while the other could be {(Fz), (Fz, FP2), $\ldots$, (Fz, FP2, Cz ,T7, T8, F8, F7, O2, P7, FP1)}. Different sequences lead to varying channel combinations, relevant when the number of channels is less than 10. The results obtained for five random sequence of channels are shown in Fig. 6.

We further analyzed the best channel combination for the task of PGES end detection. We restricted our analysis to combinations of two channels and three channels. In Table 5, we present the results for the five best combinations as well as the worse combination, i.e., the channels yielding the lowest classification performance.

**Table 5:** AUC obtained with the best channel combinations for the task of PGES end detection

2-Channel comb.	AUC	3-Channel comb.	AUC
FP2, Fz	0.80	F7, FP2, Fz	0.83
T7, Cz	0.79	T7, FP2, Cz	0.83
T7, FP2	0.79	T7, FP2, Fz	0.83
Fz, Cz	0.78	F7, T7, Cz	0.82
T8, Cz	0.78	F7, Fz, Cz	0.81
P7, P8	0.67	P7, P8, T8	0.69

The worse combination is reported in the last row

## Discussion

In this work, we investigated the feasibility of automatically detecting the end of PGES using EEG signal features. The detection of the end of PGES had been identified to be challenging in previous works, complicated mostly due to noise and artifacts. We developed a classification framework that uses time-series based features to identify if a given EEG snippet contains the end of PGES. The results from this classification framework ranked second in the official competition. One of the features used in our classification framework is inter-channel correlation, i.e. the correlation coefficient between the signal in EEG channel pairs. Transition from PGES to EEG activity will likely appear as a major event in most of the EEG channels. Other brain activities will also appear in the EEG channels and impact the correlation. However the event of EEG transition from suppression to onset of activity is expected to have a relatively larger impact in the signal level. Hence, a higher correlation could be expected for the EEG snippets consisting of an end of PGES. This could be seen to a certain extent from the feature distribution (Fig. 4) of the correlation features for the two classes: snippets with and without the PGES end. The average correlation coefficient was found to be higher for snippets that contained the end of PGES compared to those without PGES end (0.16 vs 0.10). On an individual channel level, the distribution of features for snippets with the end of PGES was shifted to the right for most of the cases (Fig. 4). This shift to the right was more pronounced for correlation features with channels in the frontal region (FP1, F7, FP2, F8) compared to other regions. This could be because all the channels in the frontal region capture the PGES transition in a similar way. Apart from the correlation features that consider the inter-channel relations, we included other features like temporal signal ratio characterizing the intra-channel dynamics. These intra-channel features were included to capture temporal changes in EEG states (transition from PGES to onset of activity) over time.

With all the inter-channel and intra-channel features used in our classifier, we obtained an ROC AUC of 0.84 (Fig. 5), significantly outperforming a random classification (AUC of 0.5). This result establishes the feasibility of detecting the end of PGES using features derived from EEG time-series In attaining this performance, it was of paramount importance to acknowledge the presence of noise and artifacts. More precisely, we used a bandpass filter to suppress noise outside of our frequency band of interest. Even such a simple noise filtering was found to be beneficial for the classification task. Analyzing with a subset of features, we found that the noise filter itself contributed to an improvement of AUC by 0.07 (Table 3). It is worthwhile to investigate other EEG denoising and artifact reduction techniques [28–31] for further improvements. This is left as future work.

We used ROC AUC as the primary evaluation metric in our work. However, it has been suggested in [21] that time distance-based evaluation metric rather than segment-based classification metric (such as ROC AUC) could be more meaningful for the task of PGES end detection. The authors used positive prediction rate of identifying an end of PGES within 5 s tolerance period as the time distance-based evaluation metric. While the work in [21] used longer signal recordings (5 min) which allow meaningful time distance-based analysis, the signal recordings in our dataset are comparatively shorter (average duration of $46.90 \pm 33.04$ s for sequences in the training set). Thus performance evaluation using time distance-based evaluation metric will be pursued in future work with longer signal recordings, as we make further improvements in the segment classification task.

PGES is associated mostly with generalized seizures [11, 32]. While focal seizures have their origin in a side of the brain (e.g. left or right), generalized effects like PGES would have their presence observable from most of the regions of the brain. Indeed, the results in Table 4 confirm that PGES is a generalized effect, since high classification performance was obtained even when only the EEG channels for specific regions of the brain were considered. Had PGES (and transition from PGES) been a localized effect, it would have been likely that the EEG channels in some regions of the brain would have yielded a degraded classification performance. However that was not observed in our results, thus confirming that PGES is a generalized effect. Interestingly, the central channels gave the best classification results compared to the left and right channel group. Only two channels are present in the central channel group while both the left and the right channel group consisted of four channels each. The central channel group (Fz and Cz) indeed contained more information relevant for PGES. In contrast, the other two-channel group (diametrically two opposite channels FP1 and P8) yielded significantly lower classification results.

We also evaluated all the possible two-channel combinations for the classification task. High variation in classification performance was seen, with highest classification results being AUC of 0.80 while the lowest AUC was 0.67 (Table 5). The best channel combinations contained at least one of the central channels (Fz or Cz), consistent with our observation that the central channels were most important for the classification task. This observation also holds true for the analysis with three-channel combinations. The signal in the parietal channels were least discriminative for the detection of the end of PGES. That the frontal and central channels provide more information for the classification could be due to the underlying physiology of PGES transitions or a mere effect of signal recording quality in different channels. For example, the physiological basis could be established if the activities in the upper brainstem, which is mostly related to PGES [18], is found to be best captured by the central and frontal channels. Similarly, noise analysis in each channel can unveil if the difference in classification results is a direct consequence of signal quality. These issues will be investigated further in future work. Nonetheless, for the task of PGES end detection, the use of all the EEG channels in the classification task was found to be important. Independent of the starting sequence of channels, the performance improved as more channels were included for classification (Fig. 6). These observations can be summarized as follows. For the task of PGES end detection, frontal and central EEG channels provide the best classification results. However inclusion of all of the available channels provide incremental improvements such that the best classification results are obtained when all the channels are included. This could be due to the generalized nature of PGES; when all channels are included then full spatial representation of PGES is captured even if good representation from some of the channels could be limited due to noise or local physiological effects.

## Conclusion

Postictal generalized EEG suppression (PGES) has been considered as one of the potential biomarkers for SUDEP risk. Accurately detecting the end of PGES, crucial for proper assessment of PGES, has been identified as challenging by previous studies. In this work, we developed a classification framework for detecting the end of PGES using time-series based EEG signal features. Our classification results establish the basic feasibility of automatically detecting the end of PGES for the downstream task of PGES characterization and SUDEP risk assessment. Owing to the generalized nature of EEG suppression in PGES, multi-channel representation was found to be important for better classification results. Signals from the central EEG channels provided the most discriminative features for PGES end detection. Further investigations of EEG signal features and classification models will be pursued as future work. A potential avenue for such investigation is a deep learning classification framework, which has recently provided encouraging results in other EEG classification tasks. We envision machine learning based synthesization of EEG signals can serve as a useful physio-marker for PGES detection/prediction to assist clinicians.

## Data Availability

The data used in this work consists of protected health information and thus were not made publicly available.
